# Adaptive Aggregate Stereo Matching Network with Depth Map Super-Resolution

**DOI:** 10.3390/s22124548

**Published:** 2022-06-16

**Authors:** Botao Liu, Kai Chen, Sheng-Lung Peng, Ming Zhao

**Affiliations:** 1 School of Computer Science, Yangtze University, Jingzhou 434023, China; liubotao920@163.com (B.L.); hitmzhao@gmail.com (M.Z.); 2 Department of Creative Technologies and Product Design, National Taipei University of Business, Taipei 10051, Taiwan; slpeng@ntub.edu.tw

**Keywords:** stereo matching, coarse-to-fine, depth map super-resolution, disparity regression, deep learning

## Abstract

In order to avoid the direct depth reconstruction of the original image pair and improve the accuracy of the results, we proposed a coarse-to-fine stereo matching network combining multi-level residual optimization and depth map super-resolution (ASR-Net). First, we used the u-net feature extractor to obtain the multi-scale feature pair. Second, we reconstructed global disparity in the lowest resolution. Then, we regressed the residual disparity using the higher-resolution feature pair. Finally, the lowest-resolution depth map was refined by using the disparity residual. In addition, we introduced deformable convolution and group-wise cost volume into the network to achieve adaptive cost aggregation. Further, the network uses ABPN instead of the traditional interpolation method. The network was evaluated on three datasets: scene flow, kitti2015, and kitti2012 and the experimental results showed that the speed and accuracy of our method were excellent. On the kitti2015 dataset, the three-pixel error converged to 2.86%, and the speed was about six times and two times that of GC-net and GWC-net.

## 1. Introduction

Visual depth estimation has its economic advantages over the lidar method. Therefore, a vision-based depth estimation algorithm has always been a popular research field. At present, the stereo matching algorithm is widely used in AR [1], MR [2], autopilot [3], robot navigation [4], and other fields.

Human acquisition of external image information is mainly based on the receptive field mechanism. The brain can infer and estimate the depth by understanding the image features captured by both eyes. Stereo matching technology lets the computer simulate this process. The main problem solved by this technology is how to find the matching pixels in the left and right view, calculate the disparity between the matching points, and finally obtain the depth map.

For example, in binocular image pairs corrected by epipolar line: we define x and y as coordinates along the width and height of the image, d as disparity, and D as depth. According to the principle of a binocular camera, if there is a pixel point (x, y) in the left image, there must be a point (x − d, y) in the right image to match the left. When we obtain the disparity, we can obtain the actual depth (D = (fb/d)) of the pixel point in the real world, where f is the focal length and b is the baseline distance of the binocular camera. However, in the application, it is vital to estimate the depth of the 3D world quickly and accurately.

In recent years, traditional stereo matching methods have been unable to effectively deal with morbid image regions such as strong reflection and low texture. In order to avoid wrong matches, an increasing number of scholars apply deep learning to stereo matching. Due to the complexity of the stereo matching algorithm, how to design a network that takes into account the stability of speed, accuracy, and robustness is our main research problem.

**Figure 1:**
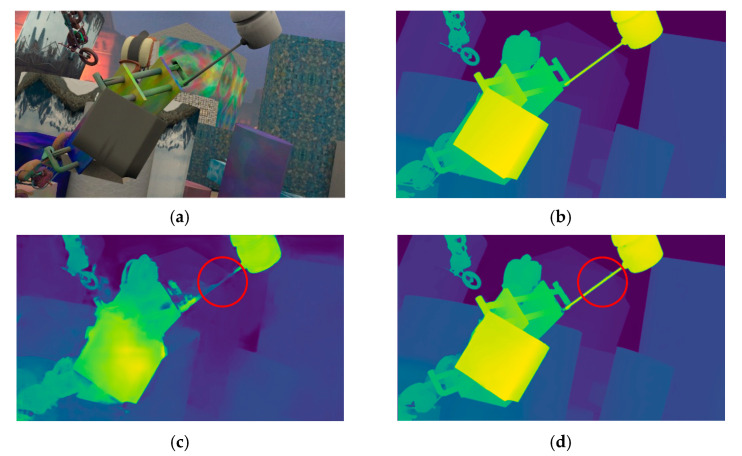
Disparity prediction results in the Scene Flow dataset; (**a**) is origin RGB data, (**b**) is Ground-Truth disparity Pseudo-color data, (**c**) is disparity prediction by stereonet, and (**d**) is our result. It can be seen from the red circle region that our method can also achieve an excellent disparity estimation in the morbid graphic area.

This paper proposes an adaptive aggregate stereo matching network with depth map super-resolution (ASR-net) based on the idea of coarse-to-fine. We adopt some ideas in Any-net [5] and GC-net [6]. However, although Any-net is fast, its accuracy is inferior. GC-net applies 3D convolution and it has received good accuracy, but the amount of calculation is too large. Our method combines their advantages and achieves a better disparity estimation, as shown in Figure 1. According to the experimental conclusion, the advantages of our network are as follows:

(1) Through a u-net multi-scale feature extractor, we only reconstructed the global depth map in the lowest resolution once. Then we refined the depth map step by step. Finally, the original depth map is restored. In this way, the calculation cost was significantly reduced.
(2) Deformable convolution and Group-wise cost volume construction were introduced into our network to thoroughly learn the global depth information of feature image pairs.
(3) Using the depth map super-resolution network ABPN [7] to replace the traditional interpolation algorithm significantly reduces the information loss in the up-sampling process.

## 2. Realated Work

The traditional representative algorithm of binocular stereo matching is SGM(Semi-Global Matching) [8,9], which obtains the disparity map through four steps: cost calculation, cost aggregation, disparity calculation, and disparity optimization [10]. Inspired by the SGM algorithm, Mayer et al. first proposed the end-to-end stereo matching network Disp-net [11], which integrates the four steps of the traditional algorithm into the neural network, and directly obtains the disparity map by inputting left–right image pairs. Kendall et al. Proposed GC-net based on Disp-net in 2017. This method introduces the concept of cost volume, constructs cost volume by splicing left and right feature images, simulates the cost aggregation of traditional methods, uses the 3D convolution method to optimize disparity [12], and finally obtains the normalized disparity map. The emergence of the GC-net laid the basic framework of a stereo matching network. Later, scholars proposed PSM-Net based on pyramid pooling [13] and GWC-net based on Group-Wise correlation [14]. In the last two years, with the rise of the attention module [15], the channel attention mechanism has been gradually introduced into the stereo-matching network. By using the channel attention module and spatial attention module to optimize the features independently, the feature information of the image is fully extracted [16,17,18]. These methods exchange better computation for a height–precision disparity map, resulting in many redundant calculations.

In order to reduce the computational cost, scholars refine the network differently. Zhang Peng et al. proposed a method based on semantic edge driving. They constructed a lightweight feature extraction module to reduce the redundancy of the feature extraction module to simplify the feature extraction steps better and improve the real-time and lightweight nature of the network [19]. In order to replace the complex 3D convolution calculation, Xu et al. proposed AA-net [20]. This method integrates multi-scale feature extraction, adaptive inter-layer aggregation (ISA), and cross-layer cost aggregation (CSA), completely replaces the 3D convolution in the current SOTA(state-of-the-art) model, and dramatically improves the reasoning speed. Khamis et al. proposed stereo-net [21] in 2018. This method is based on the idea of coarse-to-fine for the first time, using lowest-resolution images to predict depth maps and then using datasets for guided filtering to improve the accuracy of low-resolution disparity maps step by step. Based on Khamis’s research, Wang et al. proposed Any-net [5] to solve the problem that the computing power of the mobile terminal is too low to adapt to the stereo matching network. Based on stereo-net, this method uses residual optimization to restore the original resolution depth map from one-sixteenth of the input image resolution; the network saves the optimization results of each scale. The lower the accuracy of the depth map, the faster the generation speed. Finally, the depth map with different accuracy can be output according to the actual requirements. Last year, the image super-resolution task [22,23] also made a further breakthrough in depth estimation. Song et al. proposed a framework based on iterative residual learning [24]. In this method, channel attention, multi-stage fusion, weight sharing, and depth thinning were used to learn and generate a height-resolution depth map.

The above methods generally use the end-to-end network to obtain the exact size depth map output from the original size input and apply many 3D convolutions in the network layer, resulting in too much computation. Some lightweight networks usually use interpolation to up-sample low-resolution inputs [25,26,27], which causes the loss of depth information in resolution restoration. We combined the super-resolution task of depth map with a stereo matching network to solve the problem of depth information loss caused by traditional interpolation in the up-sampling part. Because the whole network only carries out global depth reconstruction for low-resolution feature image pairs, the amount of redundant calculation is significantly reduced and decreases the use of 3D convolution. Compared with other networks, our method considered both speed and accuracy and dramatically improved the generalization performance of the network.

## 3. Network Architecture

Our work focused on depth map refinement. The structure of the network refers to Any-net [5]. A lightweight u-net network is used to obtain the feature image pairs with different scales in the preliminary feature extraction process. Then, the lowest resolution features are input into the GWD-module(Group-wise cost volume with deformable convolution) for density disparity reconstruction. Then we use ABPN [7] to up-sample the depth map output from each layer. Finally, each layer’s residual module will filter the lower resolution depth map to obtain the original size depth map.

### 3.1. Features Extraction

**Figure 2:**
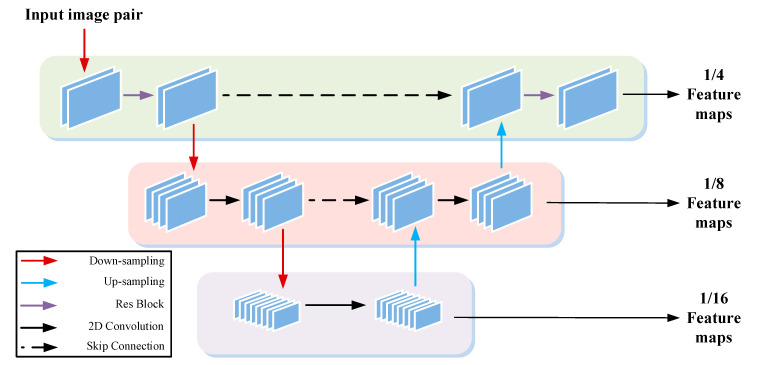
U-net feature extractor.

**Figure 3:**
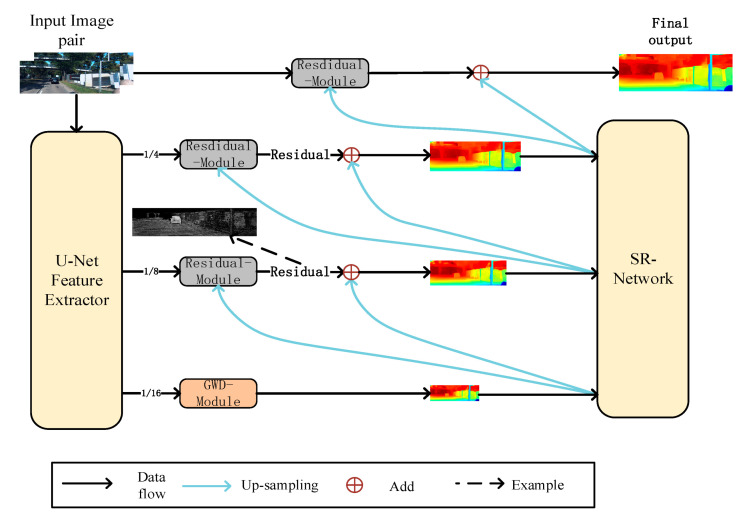
Our network‘s structure.

In order to realize the idea from coarse to fine, we used a lightweight u-net network [28,29] as the feature extractor (see Figure 2). The network structure is shown in Figure 3:

The feature extractor took left and right camera images as the input. In the process of the first layer, an 8-layer res-block [30] with dilated convolution [31] was used to extract edge features from binocular image pair. The subsequent layer used maximum pooling and stride convolution for down sampling and then a 2D convolution filter for feature extraction. Bilinear interpolation was used for up-sampling, and skip-connection was used between layers to prevent the loss of feature information. Finally, three scale feature image pairs of 1/4, 1/8, and 1/16 were obtained as the input parameters of the subsequent optimization process.

### 3.2. GWD-Module

**Figure 4:**
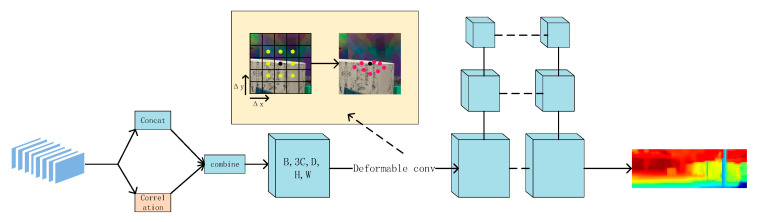
GWD Module.

The global depth reconstruction of a low-resolution feature map is the key to the whole network. In this part, we propose a GWD-module (Group-wise cost volume with deformable convolution) (see Figure 4), which includes the construction of group-wise cost volume and deformable convolution to realize adaptive cost aggregation. Finally, we used the multi-scale 3D convolution structure 3D u-net [32,33] to regularize the cost volume. Through this method, the network parameters were increased, and a better low-resolution disparity map was obtained.

#### 3.2.1. Group-Wise Correlation Cost Volume

**Figure 5:**
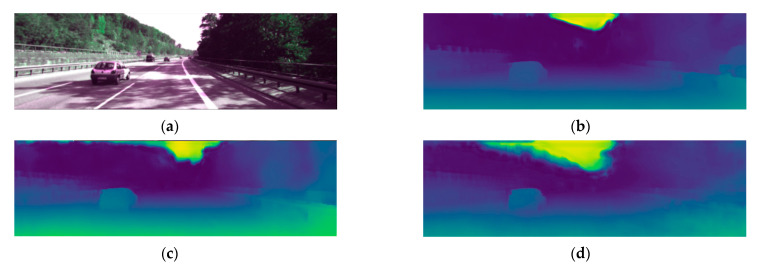
Different results with different cost volume construction methods in Stereo-net [21] ((**a**) is the real left camera’s image; (**b**) is the concatenate method; (**c**) is the inner product method; (**d**) is the subtract method).

In our research, the depth results of different cost volume construction methods have their advantages:

The concatenate method obtains the correlation between left and right images through parameter learning, while the inner product method and subtraction method estimate the correlation through artificially set measurement methods. As shown in Figure 5, the concatenate method contains more local details. The subtraction and inner product contain more global features. In order to build a cost volume that can learn more information, we combined the concatenate method and the inner product method. However, in the traditional inner product correlation calculation, we calculated and averaged all feature image pairs along the channel, which resulted in the loss of feature continuity in time and space. d means disparity level, x,y, means the width and height of feature. We denoted left unary features and right feature as $\;f_{l},f_{r}$, the number of channel is  N. 〈.,.〉 represents the inner product of two vectors, the correlation $C^{s}$ was obtained by *k*-th feature pair $f_{l}^{k},f_{r}^{k}$ for each disparity level d, as shown in formula (1):

(1) $$C^{s}(d,x,y)=\frac{1}{N}\langle f_{l}^{k}(x,y),f_{r}^{k}(x-d,y)\rangle$$

In order to deal with this problem, a group-wise correlation construction method is proposed concerning GWC-net [14], which combines the advantages of concatenation and inner product methods. Firstly, we recorded the number of feature graph channels input in the feature extractor as $N_{c}$, and divide the channels into groups $N_{k}$ along the channel dimension. Each group contains $\frac{N_{c}}{N_{k}}$ channels. The corresponding *k*-th feature group $f_{l}^{k},f_{r}^{k}$ consists of the $\;k\frac{N_{c}}{N_{k}},k\frac{N_{c}}{N_{k}}+1,...,k\frac{N_{c}}{N_{k}}+\left(\frac{N_{c}}{N_{k}}-1\right)$th channels of the original feature. The correlation calculation formula is as follows:

(2) $$C^{s}(d,x,y)=\frac{1}{N}\langle f_{l}^{k}(x,y),f_{r}^{k}(x-d,y)\rangle$$

**Figure 6:**
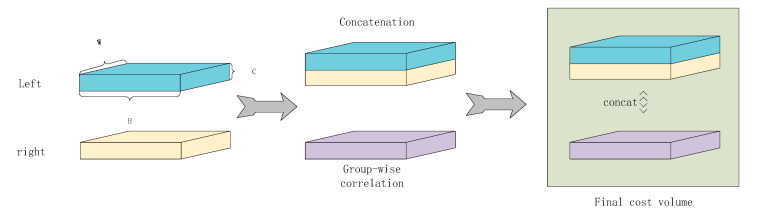
Group-wise cost volume.

In this part, the disparity candidate space is $D_{max}$. Like the input feature map, disparity candidate space must be changed to the 1/16 of the original. Finally, the shape of the cost volume is $\left[D_{max}/16,H/16,W/16,N_{k}\right]$ (see Figure 6).

#### 3.2.2. Adaptive Cost Aggregation Based on Deformable Convolution

The disparity search window is a regular rectangle in the traditional cost aggregation process. This kind of search method will cause the fusion of foreground and background information and then generate severe depth loss. In order to solve this problem, an adaptive cost aggregation based on deformation convolution is proposed. For a search window, we discerned the additional offset which consists of two bias ∆x,∆y along the width and height directions. As shown in Figure 4, this method has been used in depth map regression of AA-Net [20] and PatchMatch-Net [34] and achieves a better effect. The implementation method is shown in the formula:

(3) $${Cost}^{\prime }(d,p)=\sum_{}^{I^{2}}w_{i}*Cost(d,p+p_{i}+(△x+△y))$$

${Cost}^{\prime }\left(d,p\right)$ denotes the aggregated cost at pixel p for disparity candidata d. I is convolution kernel size, $I^{2}$ is the number of kernel‘s element (in our paper I = 3). $w_{i}$ is the aggregation weight for $I_{th}$ kernel‘s points. $p_{i}$ is the fixed offset of the standard convolution method. ∆x,∆y is additional offset. The sampling points can be qualitatively concentrated in the target area to achieve adaptive aggregation by learning and fusing these two offsets.

This step constructs three-layer convolutions to process the input cost volume. The three-layer convolutions are 1 × 1, 3 × 3, and 1 × 1. Two 1 × 1 convolutions were used to adjust the number of channels. The 3 × 3 convolution adopts the deformation convolution method and dilated convolution mode. By increasing the receptive field of the search box, the model can learn more offset information (in this paper, the expansion rate was 3).

#### 3.2.3. 3D-Unet Cost Volume Regularization

In previous work, we solved the problem of preliminary construction of cost-volume and adaptive cost aggregation. However, some noise will still be in such cost volume (such as features extracted from non-textured image areas). Although this method takes 1/16 as the minimum resolution, the lower resolution will also contain some global semantic information. Here, we constructed a multi-scale 3D-CNN model for cost volume regularization. The encoding and decoding structure is similar to u-net, which is called 3D U-net [32,33] in this paper.

Each layer uses 3D convolution, batch normalization 3D, and a Relu activation function to process the cost volume. The down-sampling process was realized by setting the convolution step size = 2, and the up-sampling uses the same 3D deconvolution layer. Skip connection was adopted between the same scale to share weight information. This regularization method makes the cost volume contain more global information. Finally, we used the softmax function to convert the correlation into probability along with the disparity candidate space of the cost volume to facilitate the subsequent error propagation.

### 3.3. Residual-Module

**Figure 7:**
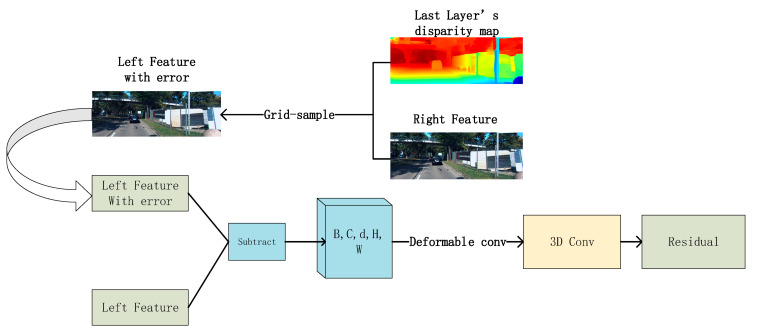
Residual module.

We used the residual module to optimize the low-resolution depth map. In order to simplify the operation, we did not carry out global reconstruction like the first layer. Firstly, we used ABPN [7] to up-sample the low-resolution depth map to make it consistent with the size of the feature map of the upper layer. Then we saved the left view generated by the correct feature map under the supervision of the low-resolution disparity map through the grid sample method in PyTorch and input it into the residual module together with the original left feature map. Finally, we obtained the residual. This item represents the error generated by the disparity map. The implementation process is shown in the following Figure 7.

Assuming there is $P_{l}\left(x,y\right)$ in left feature, the right feature can obtain match point $P_{r}\left(x+d,y\right)$ under the supervision of the disparity map. If there is no error normalization in the disparity map, these two points should be identical. Nevertheless, there must have been some wrong match in the disparity map. In order to construct the cost volume, the left feature with error is subtracted from the left feature obtained by this layer to obtain the difference. In the process of global reconstruction, our disparity candidate was 0–192, which was specified by the Kitti dataset. In the step-by-step optimization of disparity by the residual module, we set the disparity consideration as (−3,3), a total of seven possible candidates. The new disparity consideration represents the range space of error in the generated residual. In this way, the constructed cost volume is reduced from D = 192 to d = 7. It dramatically improves operational efficiency.

In this part, we still used deformable convolution. In the lowest-resolution feature of 3.2, deformable convolution is mainly used to distinguish the front and back scenes of the image. In the process of residual optimization, with the improvement of resolution, ill-conditioned graphic areas (low-textured, specular, reflective regions) will gradually appear. According to the empirical analysis of traditional cost aggregation, the error will focus on these areas. At this time, the residual cost volume constructed by deformation convolution can adaptively aggregate the ill-conditioned region error to improve the accuracy of the final result.

### 3.4. Super-Resolution Network

In the residual optimization network, the output of each scale needs to be up-sampled before error regression with a height-resolution feature map. Traditional up-sampling methods, such as bicubic interpolation, will inevitably cause information loss for depth maps with dense information. In order to optimize the original disparity map and eliminate the error caused by the traditional sampling method as much as possible, in this paper, the ABPN model was used to replace the traditional sampling method to improve the resolution of the depth map. SAB (Spatial Attention Blocks) and RBPB (Refined Back Projection Block) is proposed in this model. In the super-resolution task of the depth map, the restoration accuracy of the ABPN is much higher than that of traditional methods such as bicubic interpolation.

### 3.5. Loss Function

Our training model adopted smooth Huber loss (L1 loss) in the residual module and MDS module. In the disparity regression task, compared with L2 loss, in the disparity discontinuous region, the loss is proved to be robust, and *L*1 can significantly suppress the influence of noise. The formula is as follows:

(4) $$smooth_{L1}(x)=\{\begin{array}{c} 0.5x^{2},if\;|x|<1\\ |x|-0.5,otherwise \end{array}$$

The loss calculation for each pixel is as follows:

(5) $$L(d,\hat{d})=\frac{1}{N}\sum_{i=1}^{N}smooth_{L1}(d_{i}-\hat{d_{i}})$$

N represents the total number of marked pixels, $d_{i}$ is the actual disparity value of the GT (ground-truth) image, $\hat{d_{i}}$ is the predicted disparity value. This method corresponds to four output losses on four layers. According to the parameter quantity of each layer, we set the weight of loss for each layer as: ∂1,∂2,∂3, ∂4 = 0.25, 0.5, 1, 1 respectively. The final loss function combines the prediction results of all layers, $L_{i}$ means the value of loss. The formula is as follows:

(6) $$L=\sum_{i=1}^{layer}\partial_{i}•L_{i}$$

## 4. Experimental Evaluation

**Figure 8:**
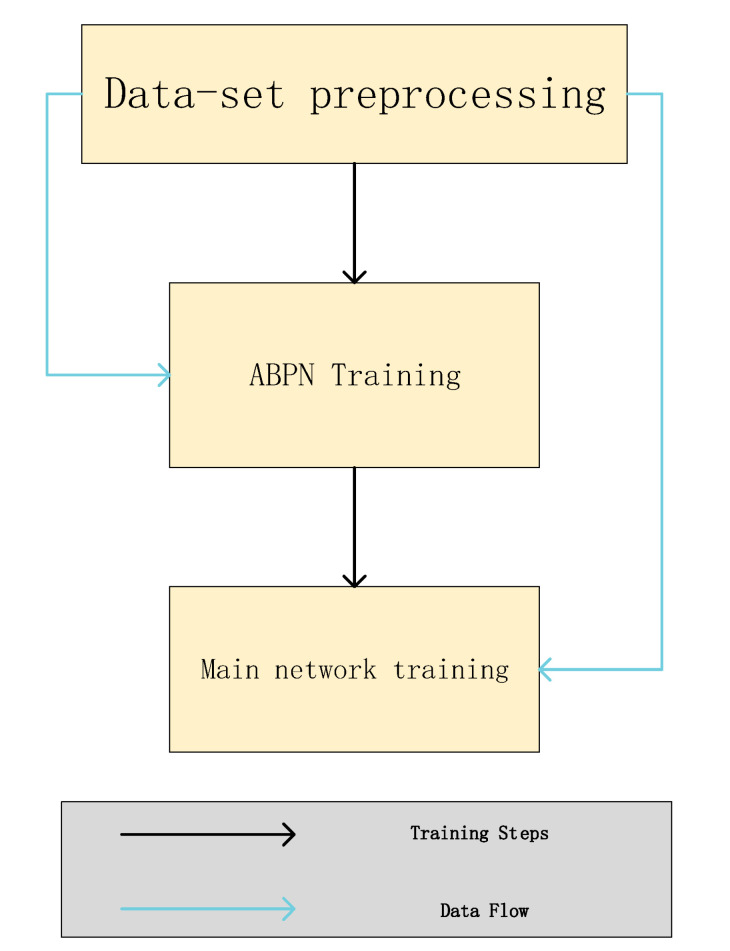
The flow chart of the experimental procedure.

The generation result of the super-resolution network ABPN will affect the generation efficiency of the primary network. Therefore, this model divides the training into two parts, and the training process is shown in Figure 8 below:

### 4.1. Dataset Preprocessing and Setup

We used PyTroch + Ubuntu to complete our two training, both using Adam (β1 = 0.9, β2 = 0.999) as our optimizer. Our experimental configuration is:

Memory: 32 GB,

CPU: AMD Ryzen9 5900X 12-core processor@3.69GHz, 

GPU: NVIDIA RTX3090 (24 GB).

(1) scene flow [11], (2) Kitti 2012 [35], (3) Kitti 2015 [35], (4) Middlebury 2014 [36] were used in our experiment. We extracted subsets from the four datasets for ABPN expansion data. Among them, (1), (2), and (3) were used in the training of the main network. The dataset is described as follows:

Scene Flow [11]: A large-scale synthetic dataset containing three sub-sets, FlyingThings3D, Monkaa, and Driving; contains daily supplies flying along random 3D paths, animated short films, and vehicular driving images similar to the KITTI dataset. Datasets provide a complete disparity image as ground truth. There are 35,454 training images and 4370 test images in the dataset, H = 540 and W = 960.

KITTI2012 [35]: This dataset was obtained by street photography of the Kitti data acquisition platform, which includes two gray cameras, two RGB cameras, a lidar, four optical lenses, and a GPS navigation system. The data includes vehicles, road signs, and other road scenes. It contains 194 pairs of stereo images with ground truth for training and 195 pairs of stereo images without ground truth for testing, H = 376, and W = 1240.

KITTI2015 [35]: KITTI2015 is similar to KITTI2012. The dataset contains 200 pairs of stereo images with ground truth that can be used for training and 200 pairs of stereo images without ground truth for testing, H = 376, and W = 1240.

Middlebury 2014 [36]: These 33 datasets were created by Nera Nesic, Porter Westling, Xi Wang, York Kitajima, Greg Krathwohl, and Daniel Scharstein at Middlebury College during 2011 to 2013. Each dataset consists of two views taken under several different illuminations and exposures. The full size of the image is H = 1988, width = 2964.

### 4.2. ABPN Training

ABPN network provides a pre-training model. However, it is necessary to use depth map datasets to fine-tune the pre-training model. We selected 200 GT depth maps from each of the three subsets of the scene flow dataset and combined all GT depth maps of kitti2015, kitti2012, and Middlebury to form a training set with a size of 1000. We first converted the PFM file in the obtained dataset into PNG format through the script. Then we constructed low-resolution datasets according to the down-sampling strategy of DVS [37] and AIR [24]. Datasets were divided into various resolutions (1/4, 1/8, 1/16), corresponding to the scale of the primary network.

**Figure 9:**
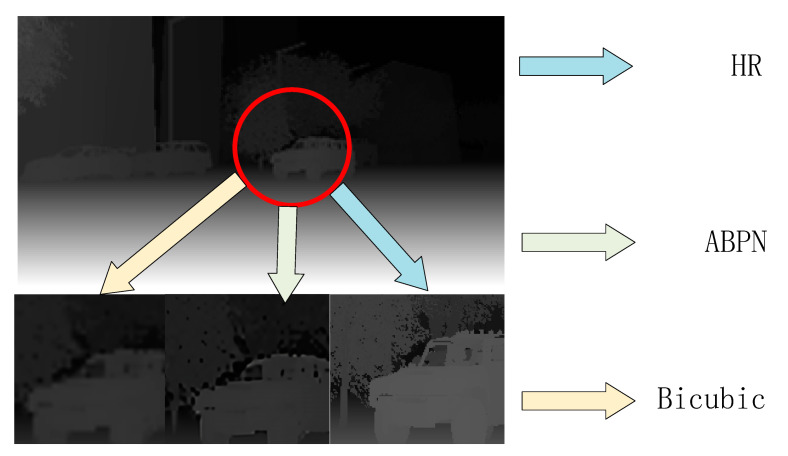
Visual comparison of different SR approaches on scene flow datasets for 4× enlargement.

The learning rate interval was set from 0.01 to 0.0001. According to the attenuation rate of 0.1, it gradually decreases with training progress (every 50 epochs), and the model converges to 300 epochs. The training time of three scales on RTX 3090 GPU is 7 h, 10 h, and 14 h. After the training, we randomly selected 100 samples from four datasets in 4.1 as the test set. The comparison results between the output results of ABPN and the traditional bicubic interpolation are as follows:

As shown in Figure 9 and Table 1, the recovery effect of ABPN on multi-scale was more accurate than that of the traditional methods. Using ABPN instead of the traditional interpolation method to carry out the up-sampling step in the primary network can further avoid the loss of depth information.

### 4.3. Main Network Training

After the training of the ABPN network, we saved and integrated the model into callable functions. In the pre-training process of the primary network, we used all the training sets of Sceneflow (35,454) and verified them on all the training sets (4370). The original data were cut randomly before training. We set the batch size as the limit value of 8, the learning rate starts at 0.001, and 30 epochs were trained. After 20 epochs, the online learning rate decreased to 0.0001. In the fine-tuning process, the mixed datasets of kitti2012 and kitti2015 were used to train 800 cycles. After 400 epochs learning rate was initially set to 0.001, and the learning rate decreased to half of the original every 100 epochs. For all datasets, the input images were normalized with ImageNet mean and standard deviation statistics. The network pre-training time is 23 h, and the fine-tune training time is 9 h.

### 4.4. Ablation Experiments

In order to verify the effectiveness of various modules proposed in this method, we tested the model through ablation experiments using Sceneflow and kitti2015 validation sets. The evaluation index of this section adopts pixel error as the experimental evaluation index. We set a threshold to judge depth estimation accuracy when the absolute difference between the output and the actual disparity is bigger than the threshold. This pixel is considered the wrong pixel. Pixel error is the percentage of these error pixels in all pixels. EPE represents the mean value of all pixel errors between the predicted image and the real image. The three pixel error is the final test result, which is composed of D1-bg (background error evaluation index), D1-fg (foreground error evaluation index), and D1-all (global evaluation index). The lower their value, the better the effect.

#### 4.4.1. Group-Wise Cost Volume

The research in Figure 5 shows that different cost volume construction methods can have a significant impact on model parameters and learning results in the stereo-net model, and different construction methods have their unique advantages. We believe that this law is also applicable to this method. In this section, ablation experiments are conducted on the cost volume construction method in the GWD-module.

**Figure 10:**
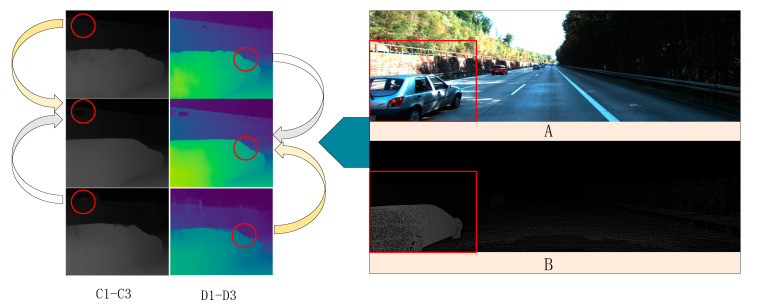
The rendering of different construction methods of cost volume. (**A**) is the left view RGB image, (**B**) is the GT depth map, we analyze the red frame area in the original image, and the red circle part represents the image area where depth recovery is difficult. (**C1**–**C3**) is the depth map generated by concatenate, ours and subtract methods, and (**D1**–**D3**) is the corresponding pseudo-color map. It can be seen that the local details of the (**C1**) image are more affluent than the others, and the accuracy is height. The accuracy of the (**C3**) image is low, but the overall image is relatively smooth. Our method combines the advantages of them.

We kept the other modules of the network unchanged. Focusing on the method of cost volume construction, we set two controls: Concatenate (left + right), the number of channels is 2C, and subtract (left − right) channels is C. Our construction method combines concatenate (left + right) and inner product <left, right>, and the number of channels is 3C. Analyze the original scale output image of the model. The evaluation index adopts EPE and three-pixel error, represents the whole image matching error, and NOC represents the unobstructed area error. It can be seen from Table 2 that the subcontract method was the fastest to build the runtime, while the concatenate method was slower than the others, but the accuracy is improved. Our method had the slowest speed, but a small amount of speed sacrifice resulted in a massive improvement in accuracy. The whole region error was about 0.28% lower than that of the subtract method, and in the whole region of the three-pixel error, the error was 0.51% lower than the subtract method. Figure 10 proved that our method fully combines the feature information of different dimensions and improves the model’s accuracy more effectively than the simple splicing of concatenation.

#### 4.4.2. Deformable Convolution

In order to explore the improvement of deformable convolution on the processing effect of image ill-conditioned areas, this section compares the model output results without deformable convolution. In Table 3, “w/o” means that deformation convolution is not used, MDS means MDS module, and Res means residual module. The results showed that on the scene flow dataset, EPE error decreased by 0.32% and 1-pixel error decreased by 3.6%. In the kitti dataset, three-pixel error D1-all decreased by 0.25%.

**Figure 11:**
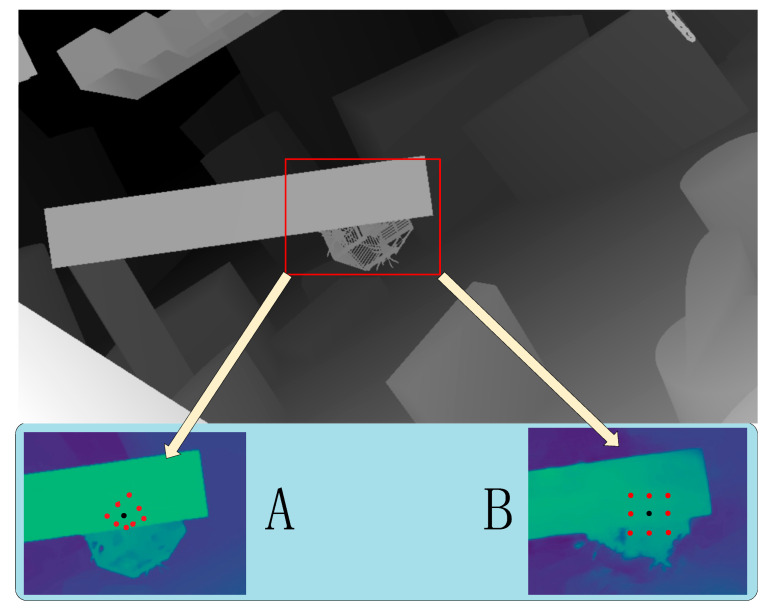
The deformable convolution ablation experiment results on scene flow (the largest figure shows the GT depth map, black dots represent the center of the convolution kernel. (**A**) is the depth map generated by this method, and (**B**) is the depth map without deformation convolution). It can be seen from the figure that the rectangle search window has caused depth misjudgment due to the integration of front and back scene information. The deformation convolution adaptively selects the same plane depth to prevent this problem.

In Figure 11A, the depth of the occluded object is restored. In the non-textured region, the depth information is smoother than that of Figure 11B. Figure 11 shows that after deformation convolution is adopted, the problems of blurring of occluded areas after reconstruction and edge loss of non-textured areas were effectively solved.

#### 4.4.3. SR-Network

**Figure 12:**
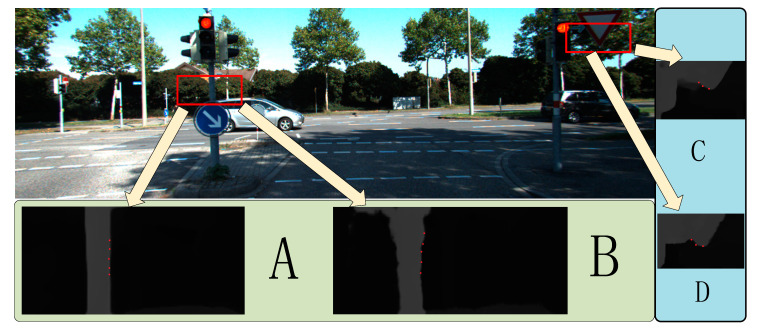
The rendering of the ABPN ablation study on Kitti2015. (**A**,**C**) show the network generation results trained with ABPN. (**B**,**D**) show the results generated by the training model with bicubic interpolation. The significant error area is marked with red dots in the figure.

In Section 4.2, the superiority of ABPN over traditional methods for sampling on depth maps was proved. In order to further prove the significant improvement of this method by introducing ABPN, the network trained by bicubic interpolation was compared with the network trained by ABPN. It can be seen from Figure 12 that in the traditional method, the most severe information loss in the sampling process is in the rod-shaped and sharp edge areas, such as the electric pole notice board. All have recovered well.

### 4.5. Results of Experiments

Finally, in order to prove the advantages of our method, it was compared with other robust stereo matching networks on two datasets:

Sceneflow: Because the dataset has a large amount of data and the accuracy of GT data is very high, it was mainly compared in terms of EPE and run-time. It can be seen from Table 4 that this method takes into account both accuracy and speed, and the error reached one-half of that of the classical stereo matching network GC-net and six times the speed.

Kitti2012, Kitti2015: For these two datasets, we used different evaluation indicators for comparison. Table 5 shows that in terms of a global error on the kitti2012 dataset, although this method was 0.28% and 0.47% higher than PSM-net and GWC-net, its speed was 2–2.5 times higher than theirs. On kitti2015 dataset, the regional error is only higher than that of PSM-net and GWC-net. It can be seen that this method takes into account both accuracy and speed, which shows that the combination of coarse-to-fine training method and complex network layer can achieve better results.

## 5. Conclusions

A real-time height-precision stereo matching network using deformable convolution and super-resolution network was proposed in our research. In this work, the problem of information adhesion caused by regular convolution in cost aggregation was avoided, and the problem of non-texture and occlusion area matching in traditional stereo matching methods was solved. At the same time, to deal with the issue of a large amount of 3D convolution calculation and many redundant parameters, this method avoids global disparity reconstruction at height resolution but in a low-resolution feature map, which significantly reduces the amount of calculation while ensuring the accuracy. This paper combined the advantages of different cost volume construction methods and adopted GWC cost volume further to improve the global semantic information of model learning. Finally, we used the super-resolution network ABPN to replace the traditional interpolation up-sampling method to reduce the information loss in primary network optimization. Compared with other networks, our experiments prove their superiority of them. Finally, we believe that there is still promotion space in the depth image super-resolution task. In future work, we will consider further improving the up-sampling process to improve the performance of our method.

## Figures and Tables

**Table 1:** Quantitative evaluation of ABPN including PSNR and SSIM for scale 4×, 8×, and 16×.

Algorithm	4×		8×		16×	
	PSNR	SSIM	PSNR	SSIM	PSNR	SSIM
Bicubic	23.27	0.603	21.35	0.544	17.76	0.434
ABPN	27.01	0.807	23.02	0.637	20.27	0.502

**Table 2:** Ablation experiment of cost volume construction method on KITTI2015.

Method	EPE (%)		3-px Error (%)		Run-Time (s)
	ALL	Noc	D1-bg	D1-All	
Concat	0.97	0.88	2.65	3.01	0.12
Subtract	1.10	1.05	2.99	3.37	0.08
Ours	0.82	0.80	2.44	2.86	0.15

**Table 3:** Ablation study of Deformable convolution.

Method	Scene Flow		KITTI 2015	
	EPE	>1 px	EPE	D1-All
w/o in MDS	1.19	12.1	0.91	2.98
w/o in Res	1.23	12.5	0.93	3.05
w/o	1.33	13.9	1.00	3.11
ASR-Net	1.01	10.3	0.84	2.86

**Table 4:** Comparison of SceneFlow EPE error.

Method	GC-Net [6]	PSMNet [13]	DispNetC [11]	GWC-Net [14]	StereoNet [19]	ASR-Net
EPE	2.52	1.09	1.68	1.12	1.10	1.01
Time (s)	0.9	0.4	0.06	0.13	0.015	0.15

**Table 5:** Performance on KITTI test set.

Method	KITTI2012		KITTI2015		Time (s)
	All	Noc	D1-bg	D1-All	
GC-Net [6]	2.30	1.77	2.21	2.87	0.9
MC-CNN [38]	3.63	2.43	2.89	3.89	67
PSMNet [13]	1.89	1.49	1.86	2.32	0.41
GWC-Net [14]	1.70	1.32	1.74	2.11	0.32
DispNetC [11]	4.65	4.11	4.32	4.34	0.06
StereoNet [19]	6.02	4.91	4.30	4.83	0.015
ASR-Net	2.66	2.17	2.35	2.86	0.15

## Data Availability

Not applicable.
